# GRecon: A Method for the Lipid Reconstitution of Membrane Proteins**

**DOI:** 10.1002/anie.201202094

**Published:** 2012-07-23

**Authors:** Thorsten Althoff, Karen M Davies, Sabrina Schulze, Friederike Joos, Werner Kühlbrandt

**Keywords:** cyclodextrins, detergents, liposomes, membrane proteins, structural biology

Membrane proteins account for about 20–30 % of the protein-encoding genes in the genomes of all living organisms.^2^ Their fundamental importance in human health and disease is underlined by the fact that many drugs in current use act on them.^3^ Functional and structural studies of membrane proteins are therefore of increasing importance but more difficult and demanding than studies with soluble proteins, because membrane proteins function in the lipid bilayer of cell membranes. For in vitro studies, the proteins are first extracted from the membrane by detergent solubilization and then purified in detergent solution. Sensitive membrane proteins are often unstable in detergent solution, but quite stable once they are reconstituted into a lipid bilayer. Moreover, many membrane proteins require a lipid environment for activity. The reconstitution process, in which detergent is replaced by lipid, must be carefully controlled, as otherwise the proteins tend to denature and aggregate.^4^

Currently, various reconstitution methods are used. They all work by reducing the concentration of the detergent below its critical micelle concentration (CMC), either by dilution, dialysis, or absorption. If detergent-solubilized lipid or preformed liposomes are present, the protein usually incorporates into the lipid bilayer. The reconstitution efficiency varies widely owing to factors that are poorly understood and difficult to control. All three methods have disadvantages. With detergents of low CMC, dialysis takes weeks, which usually results in denaturation or loss of activity of sensitive membrane proteins, especially those of eukaryotic origin. Dilution results in large volumes of low protein concentration, which makes subsequent experiments difficult, if not impossible. Detergent absorption by polystyrene beads (Bio-Beads)^5^ removes low-CMC detergents efficiently, but is often too rapid and uncontrolled, and therefore can result in protein aggregation. More recently, cyclodextrins, cyclic sugar oligomers of 950–1300 Da, have been used to remove detergents from membrane protein solutions.^6^, ^7^ Cyclodextrins work well with most commonly used detergents, but have the same disadvantage as Bio-Beads in that detergent removal can be too rapid and difficult to control. A problem common to all these methods is that they produce both proteoliposomes and empty liposomes, which ideally need to be removed. This requires another step, usually gradient centrifugation, which separates proteoliposomes by size, mass, or density.

All these conventional methods for reconstituting membrane proteins into liposomes are therefore time-consuming, labor-intensive, and unpredictable. By combining detergent removal, lipid reconstitution, and gradient centrifugation in one single step, our new GRecon (gradient reconstitution) method (Figure 1) avoids these difficulties, and produces high yields of pure, protein-containing liposomes with a wide variety of lipids, detergents, and membrane proteins.

**Figure 1:**
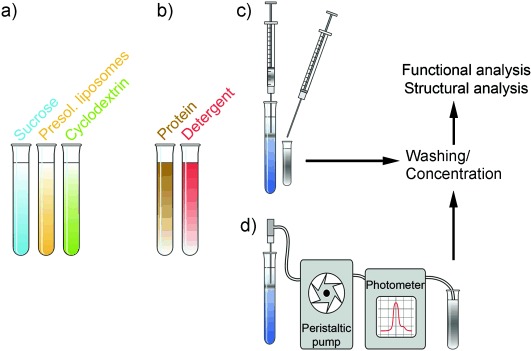
The GRecon method: a) In the GRecon gradient the concentrations of cyclodextrin and lipid increase in parallel with the sucrose density. b) The gradient is loaded with the detergent-solubilized membrane protein. As the protein migrates into the gradient during ultracentrifugation, the detergent is gradually absorbed by increasing levels of cyclodextrin, and the protein incorporates into destabilized, preformed liposomes. Opaque proteoliposome bands are harvested manually (c) or the whole gradient can be fractionated with a peristaltic pump (d). Subsequently proteoliposomes are collected by dilution and ultracentrifugation.

GRecon works with membrane proteins of all sizes and levels of complexity. It also works with a wide range of detergents, including detergents of low CMC, such as digitonin and dodecyl maltoside (DDM). DDM is a gentle detergent that is frequently used for solubilizing sensitive membrane proteins, but it is difficult to remove by dialysis because of its very low CMC of 0.01 %.^8^ Digitonin is the detergent of choice for isolating the large and fragile respiratory chain and photosynthetic supercomplexes from mitochondria, chloroplasts, and prokaryotes, which are topics of increasing interest. However, because digitonin cannot solubilize lipids effectively and its CMC is below 0.05 %,^8^ its use presents serious problems for conventional reconstitution.

We first developed the GRecon method for reconstituting the 1.7 MDa mitochondrial supercomplex I_1_III_2_IV_1_ from digitonin into proteoliposomes in order to compare its structure in a membrane environment to the three-dimensional map of the amphipol-solubilized supercomplex we determined recently by single-particle electron cryo-microscopy (cryo-EM).^9^ In the course of this work we had found that γ-cyclodextrin binds digitonin efficiently at a 1:1 molar ratio.^9^ A cyclodextrin concentration of 0.212–0.424 % (w/v) was sufficient to precipitate the supercomplex from digitonin solution, as shown by blue-native (BN) polyacrylamide gel electrophoresis (PAGE) (Figure 2 a). For single-particle cryo-EM the isolated supercomplex had been transferred into amphipols and isolated by density gradient centrifugation.^9^ Using preformed, destabilized bovine heart liposomes instead of amphipols and adding γ-cyclodextrin to the gradient as outlined in Figure 1, we succeeded in reconstituting the supercomplex into proteoliposomes in a single step by overnight ultracentrifugation.

**Figure 2:**
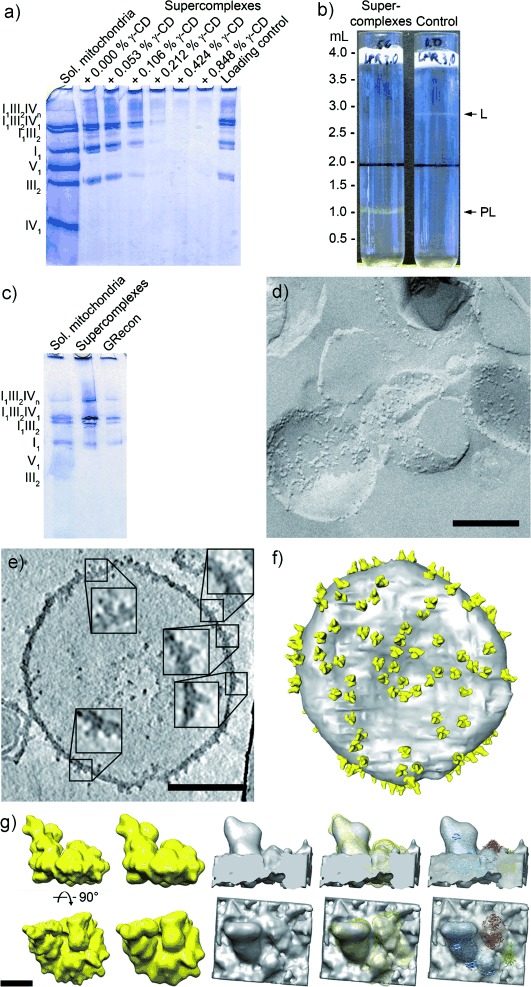
Gradient reconstitution of mitochondrial supercomplexes: a) BN-PAGE (3–10 %) of purified supercomplex I_1_III_2_IV_1_ in ca. 0.1 % digitonin after incubation with γ-cyclodextrin (γ-CD). The protein is precipitated by 0.212–0.424 % γ-cyclodextrin. b) GRecon gradients (0.3–1.3 m sucrose, 0–0.75 mg mL^−1^ bovine heart polar lipids, LPR 3 (w/w), 0–0.75 mg mL^−1^ Triton X-100, 0–0.53 % γ-cyclodextrin) with and without supercomplex. Protein incorporation causes a shift of the opaque liposome band towards higher density. L=liposomes; PL=proteoliposomes. c) BN-PAGE of resolubilized proteoliposomes. Enzymatic in-gel assay indicates the activity of complex I. d) Freeze-fracture electron microscopic image of reconstituted supercomplex. Scale bar 200 nm. e) Slice through the tomographic volume of a proteoliposome containing reconstituted supercomplexes. Inserts show twofold enlargements of individual supercomplexes. Scale bar 50 nm. f) 3D segmentation of the vesicle shown in (e) with supercomplexes in yellow. g) Comparison of the sub-tomogram average of supercomplexes from (f) and a single-particle cryo-EM map. From the left: single-particle reconstruction at 19 Å (EMD-1876), same map filtered to 65 Å, sub-tomogram average of 110 particles, sub-tomogram average with the docked single-particle map as a yellow mesh and with the docked model of the supercomplex (PDB 2ybb) with an electron density map of the enzyme from *Yarrowia lipolyti*ca.^10^ Top row: view in the plane of the membrane, bottom row: view from the matrix. Scale bar 10 nm. The matrix domains of complex I and complex III are clearly recognizable.

The opaque band containing the proteoliposomes in the lower half of the gradient (Figure 2 b) was not observed in control gradients without protein. A very small pellet of insoluble material indicated that most of the supercomplexes had been incorporated into the membrane. For biochemical analysis the opaque band was collected and diluted. Proteoliposomes were pelleted by centrifugation and resolubilized with 1 % digitonin. BN-PAGE showed the typical band pattern of supercomplex I_1_III_2_IV_1_ (Figure 2 c). In-gel activity staining demonstrated that the supercomplexes remained active throughout isolation and reconstitution. Protein incorporation was confirmed by freeze-fracture electron microscopy (Figure 2 d).

The structure of the reconstituted supercomplex in the membrane was investigated by electron cryo-tomography. Figure 2 e shows a slice through a tomographic volume, in which individual complexes are visible in the membrane. The protein densities had the characteristic l shape of complex I.^10^ In most cases, the adjacent, smaller density of the complex III dimer was also visible. Volumes of around 250 particles were averaged, resulting in a three-dimensional volume that closely resembled the map of supercomplex I_1_III_2_IV_1_, determined by single-particle cryo-EM (Figure 2 g).^9^ At an estimated resolution of 7 nm, characteristic features of the supercomplex were recognizable and the volumes of complex I and III were easily identified. The volume of complex IV did not show up clearly at this resolution, as it protrudes less from the membrane, and some of the averaged volumes may have been of the smaller supercomplex I_1_III_2_, which lacks complex IV. We also used GRecon to reconstitute purified 550 kDa ATP synthase from *Ilyobacter tartaricus* in 0.04 % DDM (Figure S1) and the 500 kDa cytochrome *bc*_1_ complex from *S. cerevisiae* in 0.05 % undecyl maltoside (Figure S2)^11^ as described in the Supporting Information.

We also applied the GRecon method to smaller, homo-oligomeric or monomeric membrane proteins. The 56 kDa carnitine transporter CaiT from *E. coli* was reconstituted into proteoliposomes for substrate-uptake studies. CaiT was prepared in 0.04 % DDM as described.^12^ A concentration of 0.182 % α-cyclodextrin, corresponding to a molar cyclodextrin/DDM ratio of 2.4:1, precipitated the protein (Figure 3 a). Linear sucrose density gradients containing 0–0.182 % α-cyclodextrin and 0–1.2 mg mL^−1^ liposomes prepared from *E. coli* polar lipids (corresponding to a lipid/protein ratio of 4:1) destabilized with 0–1.2 mg mL^−1^ Triton X-100 were loaded with detergent-solubilized CaiT. Overnight ultracentrifugation resulted in an opaque liposome band. In control gradients without protein the corresponding band had migrated less deeply into the gradient, as expected for empty liposomes (Figure 3 b). SDS-PAGE (Figure 3 c) and freeze-fracture electron microscopy (Figure 3 d) confirmed efficient CaiT incorporation. For substrate-uptake experiments with [^14^C]-l-carnitine, remaining traces of Triton X-100 were removed with Bio-Beads.^12^ Substrate uptake was saturable with an apparent *K*_M_ of (65±14) μm and a maximum capacity of (4999±320) nmol/(min mg protein) (Figure 3 e), very similar to CaiT proteoliposomes produced by detergent absorption in bulk solution.^12^

**Figure 3:**
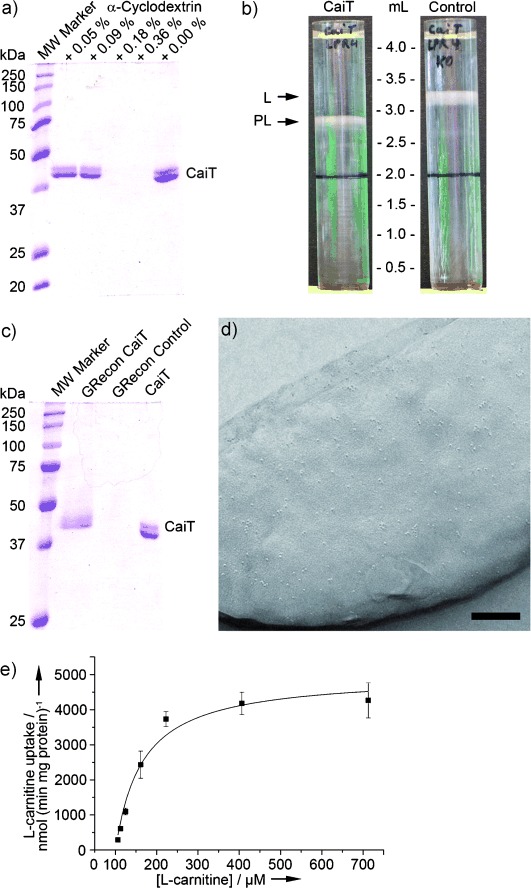
Gradient reconstitution of *E. coli* CaiT: a) α-cyclodextrin precipitates CaiT in 0.04 % DDM at a concentration of 0.182 %. b) GRecon gradients (0.3–1.0 m sucrose, 0–1.2 mg mL^−1^ *E. coli* polar lipids, LPR 4 (w/w), 0–1.2 mg mL^−1^ Triton X-100, 0–0.182 % α-cyclodextrin) with and without 500 μg CaiT after ultracentrifugation. Protein incorporation causes a shift of the opaque liposome band towards higher density. L=liposomes; PL=proteoliposomes. c) Coomassie stained SDS-PAGE of GRecon proteoliposomes indicating CaiT at roughly 43 kDa. d) Freeze-fracture electron microscopic image of GRecon CaiT proteoliposomes. Scale bar 200 nm. e) Transport kinetics of CaiT reconstituted into liposomes with GRecon.

Conventional reconstitution of CaiT into liposomes by detergent absorption had required the following steps.^12^ An aliquot of *E. coli* polar lipids in chloroform/methanol was dried under nitrogen for 3–4 h and resuspended in phosphate buffer to a final concentration of 20 mg mL^−1^ and extruded 15–20 times through a 400 nm membrane filter. The liposome solution was diluted to a final concentration of 5 mg mL^−1^ and titrated with Triton X-100 to the onset of solubilization, which was detected by light absorption at 540 nm. CaiT solubilized in 0.04 % DDM was added to a lipid/protein solution with a ratio of 20:1 (w/w). The protein–liposome mixture was incubated for 30–240 min at room temperature or at 4 °C with gentle agitation before the detergent was removed by the manual addition of a total of 450 mg of washed, blot-dried BioBeads SM-2 in batches of 50–150 mg over a period of 24–36 h. Proteoliposomes were collected by ultracentrifugation, washed, and resuspended in phosphate buffer. Each reconstitution took three days, but developing and optimizing the procedure had taken several months. With GRecon, efficient reconstitution of CaiT was achieved at the first attempt. CaiT proteoliposomes obtained by either GRecon or by conventional reconstitution were indistinguishable by freeze-fracture electron microscopy (not shown). In addition, we used GRecon to reconstitute the trimeric plant light-harvesting complex LHC-II (Figure S3) and the 32 kDa monomeric β-barrel outer-membrane porin OmpG from *E. coli* (Figure S4)^13^, ^14^ into proteoliposomes, as described in the Supporting Information.

GRecon is clearly a very valuable new tool for reconstituting membrane proteins solubilized in a wide range of detergents. It is suitable both for functional reconstitution of small membrane proteins such as CaiT, and of large, fragile multicomponent complexes such as the supercomplexes of the mitochondrial respiratory chain. SDS-PAGE and freeze-fracture electron microscopy revealed incorporation at levels that were equal to, or better than, standard protocols. Reconstitution was efficient and achieved far more easily than with conventional methods.

The success of all reconstitution protocols depends on the speed at which the detergent is removed.^4^ In the GRecon method, this process is under the control of two parameters, the cyclodextrin concentration in the gradient and the steepness of the density gradient itself, which both determine the rate at which the detergent is absorbed. The required cyclodextrin concentration is easily determined in a preliminary precipitation experiment. In most cases a final molar ratio of 1.2:1 to 2.4:1 (cyclodextrin/detergent) is sufficient.^6^ Detergent removal starts slowly and then progresses as the protein migrates into the gradient. By contrast, the rate of detergent removal by dialysis or absorption in bulk solution is highest at the outset, when the concentration of detergent monomers is at its maximum, and then decreases. This often results in protein precipitation, unless special precautions are taken, such as adding Bio-Beads manually one by one, and offering a large excess of lipids. GRecon does not require a large excess of lipids, and thus works at considerably lower lipid-to-protein ratios than other methods.^4^

The GRecon density gradient should be adapted to the size of the protein. For small membrane proteins of 30 to 150 kDa, gradients of 0.3–0.8 m sucrose work well, larger proteins up to 600 kDa require a sucrose concentration up to 1.0 m, and large complexes above 1 MDa need up to 1.3 m. The proteoliposomes obtained are equally suitable for structural and functional studies, as we have shown. If transport measurements require sealed liposomes, remaining traces of Triton X-100 can be removed with Bio-Beads.

GRecon takes advantage of the different detergent-absorbing properties of α-, β-, and γ-cyclodextrins. The latter sequesters bile-salt-derived detergents with high affinity, and is thus useful for reconstituting membrane proteins solubilized in digitonin. β-Cyclodextrin has a higher detergent affinity than α-cyclodextrin. This may be desirable in special cases, but the high affinity of β-cyclodextrin precludes the use of Triton X-100 for destabilizing preformed liposomes.^6^

In summary, GRecon offers a number of decisive advantages over conventional reconstitution procedures: 1) As a single-step method it is quick and convenient, requiring minimal manual sample handling. 2) Reconstitution works reliably and results are reproducible. 3) Successful reconstitution is immediately apparent from the shift of the opaque proteoliposome band. 4) Proteoliposomes are separated automatically from empty liposomes on the density gradient. This separation would add another overnight step to the conventional protocol, during which a significant portion of the reconstituted protein is inevitably lost. 5) The GRecon method indicates protein aggregation or denaturation by the formation of an insoluble pellet at the bottom of the gradient. In conventional protocols, which do not normally include a final gradient centrifugation step, it is difficult to separate the denatured from the reconstituted protein, and thus to be sure about the reconstitution efficiency. 6) With GRecon it is easy to try out different lipids or lipid combinations. This is important as not every protein is compatible with every lipid. By conventional methods, it can take weeks or months to find the right lipid combination.

In addition, GRecon opens up interesting new perspectives for the two-dimensional (2D) crystallization of membrane proteins. 2D crystals are needed to determine the structure and conformational dynamics of membrane proteins by electron crystallography.^8^ 2D crystallization by detergent removal with cyclodextrin is in principle an elegant and highly promising technique, although so far this approach seems to have been successful in only two cases.^7^ Out of the six GRecon-reconstituted membrane proteins reported here, two (the cytochrome *bc*_1_ complex and LHC-II, see the Supporting Information) formed 2D crystals on the gradients overnight, even though this was not even intended. If necessary, 2D crystal formation can be easily avoided by increasing the lipid/protein ratio. These results shows the great potential of GRecon for the 2D crystallization of membrane proteins, which requires systematic variation of parameters such as lipids and detergents, protein concentration, and lipid/protein ratio. With GRecon, all these parameters are easily tested, and results are obtained in minimum time.

Perhaps the most important point is that GRecon works well with detergents of low CMC, especially with digitonin which is otherwise problematic. There are only very few reports of the successful reconstitution of membrane proteins from digitonin solution in the literature.^15^ To our knowledge the reconstitution of supercomplexes into liposomes for structural or functional studies has not been achieved before. As the structures and mechanisms of more and more small and medium-sized membrane proteins are determined, these massive membrane assemblies come increasingly into focus. The reconstitution of membrane protein supercomplexes into proteoliposomes opens up new ways for investigating their molecular mechanisms, which are almost entirely unexplored.

## Experimental Section

Gradient reconstitution: Sucrose gradients were prepared in 4 mL SW60 ultracentrifuge tubes (Beckman-Coulter) on a Biocomp Gradient Master (ScienceServices, München) based on the method of Coombs and Watts.^16^ Concentrated sucrose solution (2 mL) containing Triton-destabilized preformed liposomes was layered under an equal volume of light solution (0.3 m sucrose) in protein buffer without detergent in centrifuge tubes. Liposomes were prepared by drying a film of lipid dissolved in chloroform (Avanti Polar Lipids) under a nitrogen stream and suspending in protein buffer. An equal amount (w/w) of Triton X-100 was added to destabilize the liposomes.^4^ After 30 min at RT, appropriate amounts of sucrose and cyclodextrin were added and dissolved. The liposome suspension was sonicated briefly to obtain liposomes of roughly equal size. The test tubes were closed with caps to expel all air, and gradients were formed by rotation. A 200 μL aliquot was removed from the top of the gradient before the protein sample was added. Protein portions of 500–600 μg per gradient were loaded and the gradients centrifuged at 150 000×*g* for 18:00 h. After ultracentrifugation, opaque liposome bands were collected with a syringe and diluted with detergent-free buffer to remove most of the sucrose and Triton X-100. Proteoliposomes were pelleted at 125 000×*g* for 1 hour at 4 °C and resuspended in roughly 50 μL buffer. For substrate uptake assays, Triton X-100 was removed by incubating the solution overnight at 4 °C with an excess of Bio-Beads SM-2 (Bio-Rad).

Biochemical and biophysical methods: Mitochondrial supercomplexes solubilized in digitonin were purified by ultracentrifugation into sucrose gradients supplemented with 0.1 % digitonin.^9^ Wild-type *E. coli* CaiT was expressed and purified as described.^12^ For GRecon the purified protein was diluted to 1 mg mL^−1^ in buffer with 0.04 % DDM. Incorporation of protein into liposomes was assayed by SDS-PAGE.^17^ Gels were stained with Coomassie R250.^18^ Uptake of ^14^C-labeled l-carnitine by CaiT was measured as described.^12^

Freeze-fracture electron microscopy: Incorporation of proteins into liposomes was assessed by freeze-fracture electron microscopy. Proteoliposome suspension (2–3 μL) was placed in copper holders, flash-frozen in liquid ethane and fractured at −135 °C in a Balzers (Liechtenstein) BAF 060 freeze-fracture apparatus. Surfaces were shadowed with platinum/carbon at an angle of 45° and carbon at 90°. Replicas were washed in chromic/sulfuric acid and water, and imaged in a EM208S electron microscope (FEI Eindhoven, Netherlands) equipped with a 1k×1k TVIPS CCD-camera (Tietz, München, Germany).

Electron cryo-tomography and sub-tomogram averaging: Samples of the proteoliposome suspension (2–3 μL ) were placed on Quantifoil R2/2 holey carbon film grids (Quantifoil Micro Tools, Jena, Germany), blotted, and vitrified by plunging into liquid ethane with a manual device. Tilt series ±60° were recorded in 1.5° increments under low-dose conditions in a TECNAI G2 Polara (FEI, Eindhoven, The Netherlands) equipped with a post-column 863 Tridiem energy filter (Gatan, Pleasanton, CA, USA). Images were recorded on a 2k×2k CCD (5.76 Å/pixel) at 300 kV and 7 μm underfocus. Data was processed with Etomo (IMOD, Boulder, CO)^19^ and segmented by automatic and manual procedures in Amira (Visage Imaging, Berlin, Germany). Before segmentation, contrast was enhanced using non-anisotropic diffusion.^20^ For sub-tomogram averaging, aligned images in the tilt series were filtered to 3 nm before back-projection. The locations of 250 densities of complex I from one tomogram were picked manually in 3DMOD (IMOD, Boulder, CO)^19^ and aligned against the matrix arm of the bovine heart supercomplex using PEET.^9^, ^21^ 110 particles were used for the final average, which was smoothed with a median filter (*n*=6) to remove noise. The resolution of the final volume was estimated at approximately 7 nm.

## Supplementary material

Detailed facts of importance to specialist readers are published as ”Supporting Information”. Such documents are peer-reviewed, but not copy-edited or typeset. They are made available as submitted by the authors.
