# Supplementary material for: GRecon: A Method for the Lipid Reconstitution of Membrane Proteins
Source: Angew Chem Int Ed Engl. 2012 Jul 23;51(33):8343–7. doi: 10.1002/anie.201202094 (PMC3494379; doi:10.1002/anie.201202094)
Supplement: Supplementary file 1 [file anie0051-8343-SD1.pdf]

Angewandte Chemie
Eine Zeitschrift der Gesellschaft Deutscher Chemiker

Supporting Information

© Wiley-VCH 2012

69451 Weinheim, Germany

GRecon: A Method for the Lipid Reconstitution of Membrane Proteins**

*Thorsten Althoff, Karen M. Davies, Sabrina Schulze, Friederike Joos, and Werner Kühlbrandt**

anie_201202094_sm_miscellaneous_information.pdf

Supporting Information

We tested the GRecon method with a range of other membrane proteins, including ATP-synthase, cytochrome bc_1 complex, plant light harvesting complex II, and outer membrane porin OmpG, which differ in size, complexity, oligomeric state and secondary structure.

The ATP synthase (complex V of the respiratory chain) uses an ion gradient across the membrane as energy to produce ATP. We chose the 550-kDa enzyme from *Ilyobacter tartaricus* to investigate whether this fragile multi-subunit complex could be reconstituted into proteoliposomes by the GRecon method. After the initial precipitation test with α-cyclodextrin to remove the DDM used for solubilization, large amounts of protein remained in the supernatant (Figure S1a), as the detergent had been concentrated from originally 0.04% to approximately 0.17%. Nevertheless, gradient reconstitution was successful. Gradients containing 0 – 0.389% α-cyclodextrin, a mixture of solubilized polar lipids from *E. coli* and soybean and sucrose ranging from 0.3 to 1.0 or 1.3 M contained faint bands of proteoliposomes (Figure S1b). Bands in the lower and higher density range of the gradients were assayed for incorporated protein by SDS-PAGE (Figure. S1c) and freeze-fracture EM (Figure S1d,e). Both methods indicated that only the band at higher density of the gradient contained the reconstituted ATP synthase.

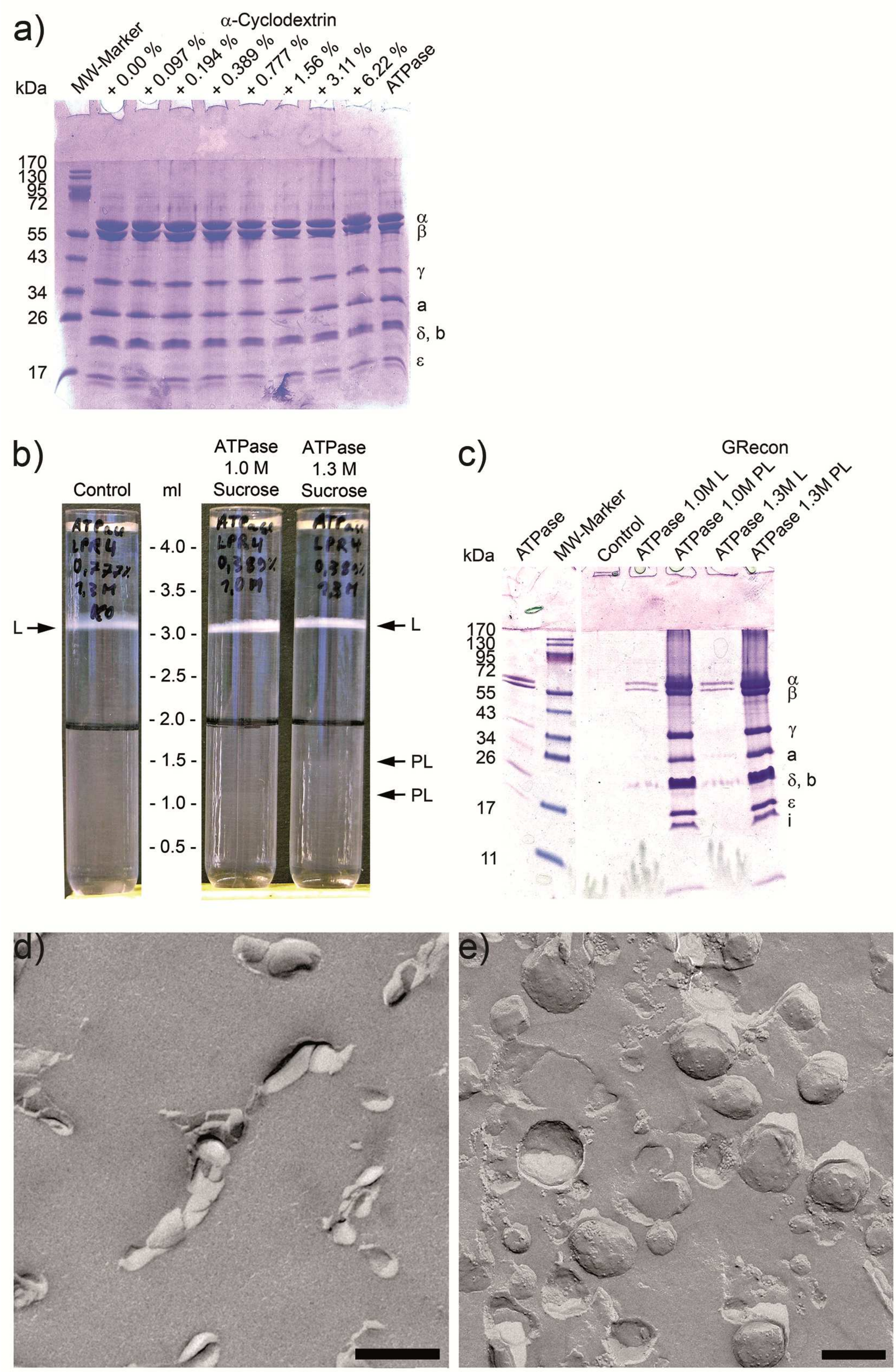

Figure S1. Gradient reconstitution of *I. tartaricus* ATP-synthase: a) Detergent removal by α-cyclodextrin from ATPase solubilized in ~0.17% DDM. Soluble protein subunits are found in the supernatant up to 6.22% cyclodextrin. b) Gradients with 0.3 – 1.0 M or 0.3 – 1.3 M sucrose, 0 – 0.389% α-cyclodextrin, 0 – 0.6 mg/ml *E. coli* polar lipids, 0 – 0.6 mg/ml soybean polar lipids (LPR4) and 0 – 1.2 mg/ml Triton X-100. 600 µg ATP-synthase was loaded per protein gradient. Faint bands of proteoliposomes (PL) are visible in the higher density range, whereas liposomes (L) are found in the upper half. c) Coomassie-

stained SDS-PAGE (13%) of gradient fractions from (b) resolubilized with 2% SDS indicating that only the lower bands contain significant amounts of ATP synthase. Black lines were drawn on the gradient tubes for reference. d) Freeze-fracture of liposomes from the L-band in the 0.3 – 1.0 M sucrose gradient in (b) indicates the absence of incorporated protein. e) Freeze-fracture micrograph of proteoliposomes (PL-band) from the same gradient shows protein has been incorporated. Scale bars 200 nm.

The 500-kDa cytochrome bc_1 complex from *S. cerevisiae* (complex III of the respiratory chain) was solubilized in 0.05% undecyl-maltoside (UDM), and isolated and purified as described.[9] The detergent appeared to be concentrated roughly by a factor of 10 in the ultrafiltration step, as even a 40-fold molar excess of α-cyclodextrin (4.72%) did not precipitate the complex completely (Figure S2a). GRecon gradients were prepared with 1.77% and 2.36% α-cyclodextrin corresponding to a 1.8- and 2.4-fold molar excess relative to the estimated UDM concentration of 0.5%, respectively. With 0 - 2.36% α-cyclodextrin in a gradient ranging from 0.3 – 0.8 M sucrose, a reddish opaque band was found in the upper half of the gradient after centrifugation. With 0 - 1.77% α-cyclodextrin in a 0.3 – 1.3 M sucrose gradient, a similar band was found in the lower half (Figure S2b). SDS-PAGE showed that both contained cytochrome bc_1 complex (Figure S2c). Freeze-fracture EM revealed that the former contained proteoliposomes with individually incorporated complexes, whereas in the latter case the complex was densely packed and formed 2D crystals (Figure S2d,e).

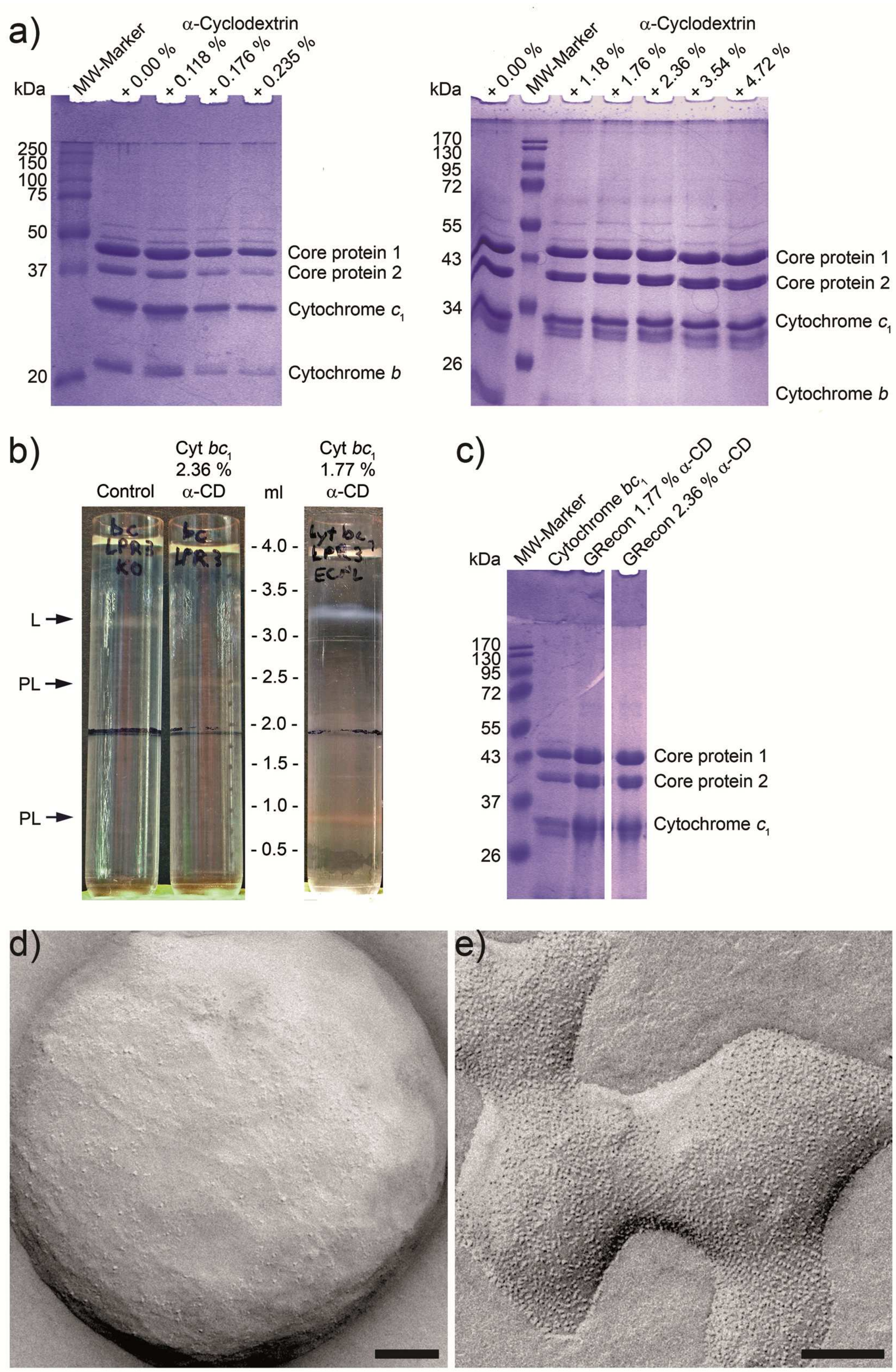

Figure S2. Gradient reconstitution of *S. cerevisiae* cytochrome bc_1 complex: a) Detergent removal by α-cyclodextrin from cytochrome bc_1 in ~0.5% UMG. Even at very high cyclodextrin concentrations the supernatant contains unprecipitated protein. b) left: control and cytochrome bc_1 gradients with 0.3 – 0.8 M sucrose and 0 – 2.36% α-cyclodextrin; right: 0.3 – 1.0 M sucrose and 0 – 1.77% α-cyclodextrin with 0 – 0.9 mg/ml *E. coli* polar lipids (LPR3), destabilized with 0 – 0.9 mg/ml Triton X-100. Samples of 600 µg cytochrome bc_1 were loaded per protein gradient. L = liposomes; PL = proteoliposomes. Black lines

were drawn on the gradient tubes for reference. c) Coomassie-stained SDS-PAGE (15%) of gradient fractions from (b) showing subunits of the bc_1 complex. d) Freeze-fracture electron micrograph of GRecon-reconstituted cytochrome bc_1 from the 0.3 – 0.8 M sucrose gradient containing 0 – 2.36% α-cyclodextrin. Proteoliposomes contain individual, well-separated complexes. e) Freeze-fracture EM of the sample from the gradient with 0.3 – 1.0 M sucrose and 0 – 1.77% α-cyclodextrin, showing the formation of 2D crystals. Scale bars 200 nm.

The trimeric light harvesting chlorophyll a/b protein complex II (LHC-II) from pea (*Pisum sativum*), which has a total mass ~150 kDa, was used as a membrane protein of intermediate size. LHC-II was solubilized in 1% NG.[11] Titration with α-cyclodextrin revealed that a 1.2:1 molar ratio or 3.81% of cyclodextrin was sufficient to remove all detergent and precipitate the protein (Figure S3a). GRecon gradients were prepared with 0.3 – 1.3 M sucrose, 0 - 0.57% α-cyclodextrin and thylakoid lipids (50% MGDG, 31% DGDG, 10.7% PG and 8.3% SQDG) solubilized in Triton X-100 at different lipid-to-protein ratios. After ultracentrifugation, a strong dark green band was found in the lower quarter of the gradient at LPR 15. At LPR 5 there was a dark-green pellet. Both gradients also showed a green band near the top (Figure S3c). The light green color of the gradients loaded with protein is due to free chlorophyll released by detergent. SDS-PAGE indicated that the band from the higher gradient density and the pellet contained significant amounts of LHC-II (Figure S3b). Freeze fracture analysis revealed that the proteoliposome band from the gradient with LPR 15 contained vesicles densely packed with proteins, mostly in 2D crystal lattices (Figure S3d). The pellet from the gradient at LPR 5 consisted entirely of 2D crystals (Figure S3f). The liposome band consisted mostly of small empty vesicles and only very few small patches of 2D crystals (Figure S3e). These had most likely been trapped by the empty vesicles and thus did not migrate into the gradient. Note that LHC-II formed 2D crystals on all GRecon gradients we tested. The 2D arrays of the cytochrome bc_1 complex and LHC-II clearly demonstrate potential of the new GRecon method for 2D crystallization of membrane proteins.

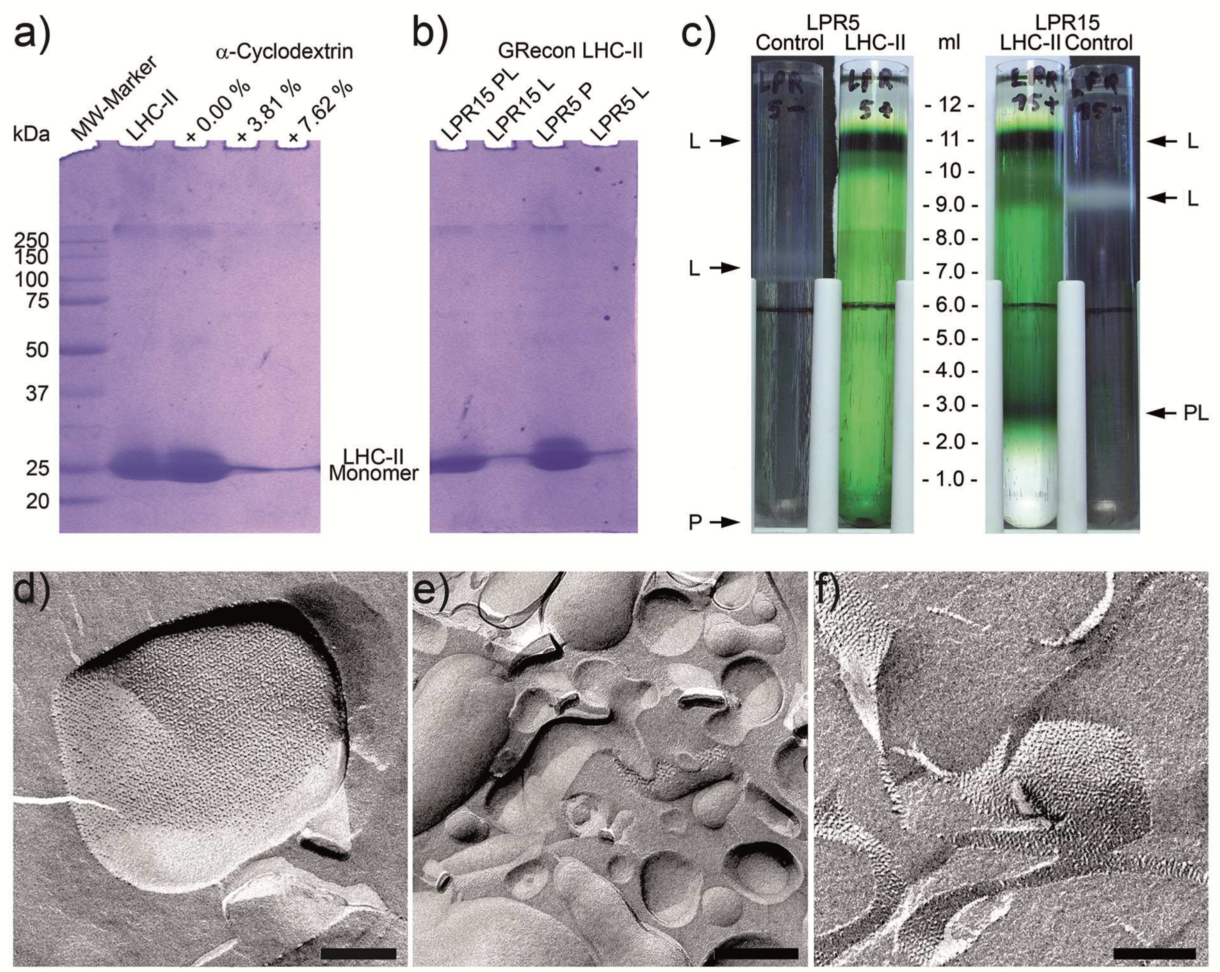

Figure S3. Gradient reconstitution of *P. sativum* LHC-II: a) Removal of 1% NG by α-cyclodextrin from solubilized LHC-II. At 3.81% cyclodextrin (1.2-fold molar excess) very little LHC-II remains in the supernatant. b) Coomassie-stained SDS-PAGE (12%) of gradient fractions from (c) resolubilized with 1% NG. LHC-II is present in the liposome band and pellet. c) left: protein and control gradients with 0.3 – 1.3 M sucrose and 0 – 0.57% α-cyclodextrin supplemented with 0 – 0.35 mg/ml thylakoid lipids and 0 – 0.29 mg/ml Triton X-100 (LPR 5); right: 0 – 1.05 mg/ml thylakoid lipids and 0 – 0.87 mg/ml Triton X-100 (LPR 15). Protein gradients were loaded with 1 mg LHC-II. L = liposomes; PL = proteoliposomes; P = pellet. Black lines were drawn on the gradient tubes for reference. d) Freeze-fracture electron micrographs of proteoliposomes from the PL-band of the LPR15 gradient in (c) shows densely packed proteins at the verge of 2D crystallization. e) Freeze fracture EM shows that the L-band from the same gradient consists almost entirely of small liposomes, with an occasional trapped 2D crystal. f) The pellet from the LPR5 gradient consists largely of 2D crystalline sheets. Scale bars 200 nm.

Finally, we tried the small, monomeric 32-kDa β–barrel outer membrane porin OmpG from *E. coli*. OmpG was expressed and purified as described,[12] except that a construct with 15 additional amino acids, including 6 N-terminal histidines, was used and the detergent was exchanged to 0.4% NG during the second ion exchange chromatography step. The initial precipitation test worked with 1.52% α-cyclodextrin (1.2:1 molar ratio). In this case the protein not only precipitated but unfolded, as indicated by a shift of the band in SDS-PAGE (Figure S4a). After ultracentrifugation of OmpG into a density

gradient containing 0.3 – 0.8 M sucrose, *E. coli* polar lipid at LPR 4 (destabilized as before with Triton X-100) and 1.52% α-cyclodextrin, opaque liposome bands were visible both in the protein-containing and the control gradient. In the former the proteoliposomes had migrated deeper into the gradient (Figure S4b). SDS-PAGE showed a band at 32 kDa, indicating that the OmpG in the liposomes was correctly folded, although some OmpG unfolded during SDS-PAGE (see controls; Figure S4c). Freeze-fracture electron microscopy confirmed that a large number of OmpG monomers had been incorporated into the liposomes (Figure S4d). Reconstitution of OmpG into proteoliposomes by conventional methods was rarely successful for unknown reasons.

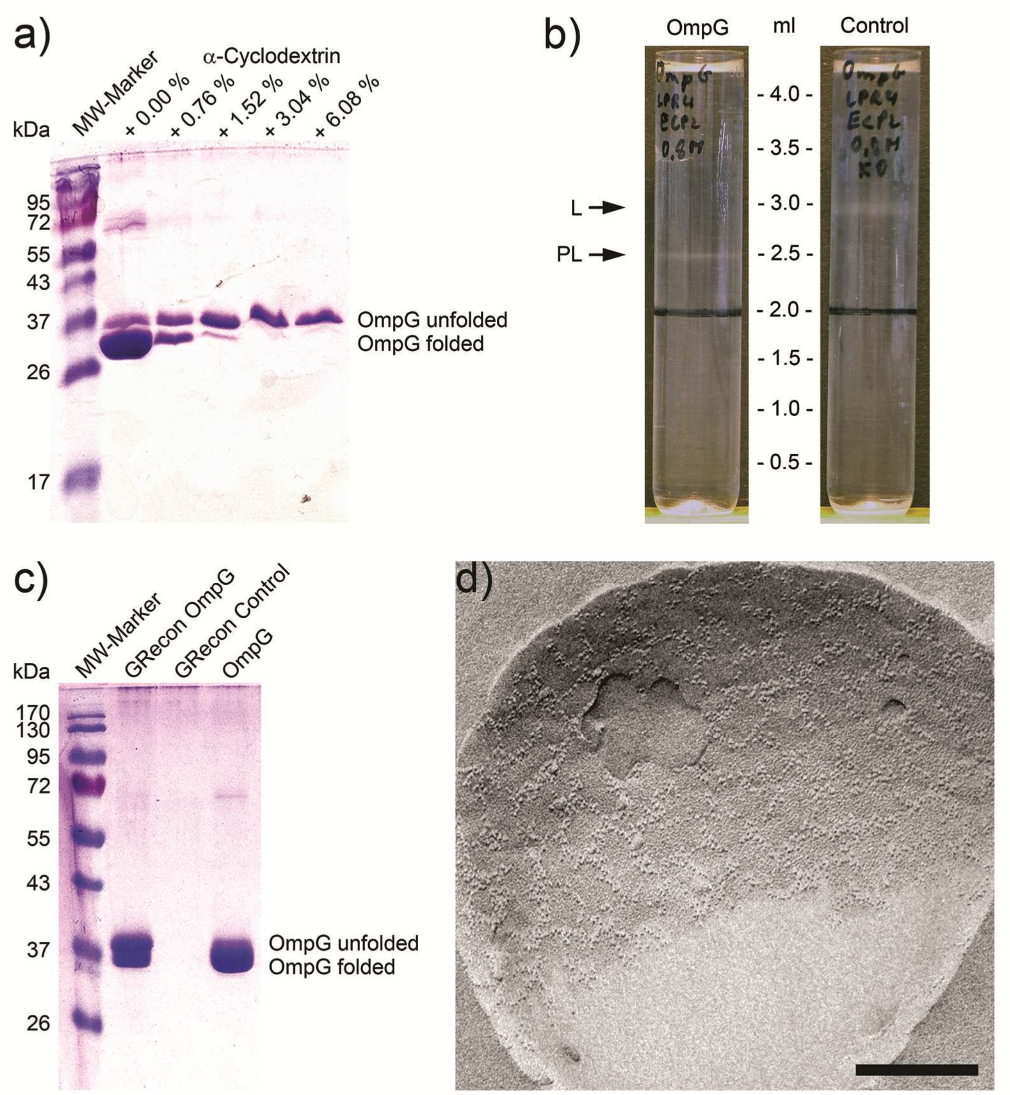

Figure S4. Gradient reconstitution of *E. coli* OmpG: a) Detergent removal by α-cyclodextrin from OmpG solubilized in 0.4% NG. Above a cyclodextrin concentration of 1.52% the protein is mostly unfolded. b) Gradients with 0.3 – 0.8 M sucrose, 0 – 1.52% α-cyclodextrin, 0 – 1.2 mg/ml *E. coli* polar lipids (LPR4) and 0 – 1.2 mg/ml Triton X-100. 600 µg OmpG were loaded per protein gradient. L = liposomes; PL = proteoliposomes. Black lines were drawn on the gradient tubes for reference. c) Coomassie-stained SDS-PAGE (15%) of gradient fractions from (b) showing mostly folded protein in the liposome band collected from the gradient. d) Freeze-fracture electron microscopy of GRecon-reconstituted OmpG. Scale bar 200 nm.
